# Alcohol consumption and breast lesions: targets for risk-based screening in high-risk Italian women

**DOI:** 10.1007/s12282-025-01720-8

**Published:** 2025-05-16

**Authors:** Sonia Cerrai, Alessio Lachi, Michela Franchini, Stefania Pieroni, Giada Anastasi, Marco Scalese, Anna Odone, Silvano Gallus, Luc Smits, Sabrina Molinaro

**Affiliations:** 1 Epidemiology and Health Research Lab, Institute of Clinical Physiology of the National Research Council of Italy (IFC-CNR), Pisa, Italy; 2 Department of Epidemiology, Care and Public Health Research Institute (CAPHRI), Maastricht University, Maastricht, The Netherlands; 3 Saint Camillus International University of Health and Medical Sciences, Rome, Italy; 4 Institute of Computer Science, University of Pisa, Pisa, Italy; 5 Department of Public Health, Experimental and Forensic Medicine, University of Pavia, Pavia, Italy; 6 Department of Medical Epidemiology, Istituto Di Ricerche Farmacologiche Mario Negri IRCCS, Milan, Italy

**Keywords:** Breast lesion, Alcohol, Risk-based screening, Event History Analysis

## Abstract

**Background:**

Breast cancer in Italy is still the most frequent cancer among women, and alcohol consumption is recognized as a risk factor for its development. Overall, in 2020, approximately 10% of all breast cancer-related deaths were attributable to alcohol consumption. Despite advancements in diagnostics and therapeutic options reducing mortality trends, the incidence of breast cancer is projected to rise in Italy. This study aims to assess how alcohol consumption influences the timing of breast lesion diagnosis. Understanding these associations can enhance primary prevention strategies and support the adoption of a risk-based prevention approach, integrating lifestyle factors into personalized screening programs.

**Methods:**

P.I.N.K. (Prevention, Imaging, Network and Knowledge) study collected data on a prospective dynamic cohort of women who voluntarily underwent breast cancer screening at breast centers throughout Italy, between 2018 and 2023, outside the free national screening program. The occurrence of breast lesion diagnosis and baseline information were collected through clinical visits and an auto-administered questionnaire, including data on absent, moderate or high alcohol consumption during the last 12 months and smoking. 3774 women (mean age 58.9 ± 10.0, range 40–98 years) were included in the present analysis, encompassing women with a suspected or confirmed diagnosis of benign or malignant tumor and healthy women that contributed at least 4 years to the study. An Event History Analysis was carried out to evaluate the effect of alcohol consumption on the timing to event. The event was represented by the transition of the health status, from not diagnosed to diagnosed with breast lesion. The Accelerated Failure Time parameterization was used to directly interpret how the covariates influence the time to the event. The model was adjusted by familiality of breast/ovarian cancer, marital status, level of education, and type of access to health care.

**Results:**

High alcohol consumption exhibited an accelerating effect on the transition to the diagnosed state, indicating a significantly shortened time to event: *β* coefficient − 0.33 (*p*-value 0.010) in the adjusted model, indicating an anticipation of about 4 months. The effect of moderate alcohol consumption did not reach statistical significance, neither in the unadjusted model nor in the adjusted model. Adjustment for smoking status led to a further increase of the *β* coefficient for high alcohol consumption (− 0.40; *p* value 0.003) and brought moderate alcohol consumption closer to statistical significance (*β* − 0.15; *p*-value 0.087). Familiality of breast or ovarian cancer showed a statistically non-significant accelerating effect, while marital status different from maiden, high education, and private access to health care showed decelerating effects.

**Conclusions:**

High alcohol consumption was confirmed as an accelerating factor in breast lesions diagnosis, while the effect of moderate consumption did not reach statistical significance. These results help identifying actionable targets for high-risk populations, emphasizing personalized risk-based screening programs and gender-sensitive interventions.

**Supplementary Information:**

The online version contains supplementary material available at 10.1007/s12282-025-01720-8.

## Background

### Impact of breast cancer

Globally, breast cancer (BC) significantly impacts women’s health, contributing to substantial suffering and premature mortality [1]. According to the World Health Organization (WHO) and the Global Cancer Observatory, BC is the most common cancer among women worldwide, with 5 diagnoses per 1,000 women and approximately 670,000 deaths in 2022 (www.who.int/news-room/fact-sheets/detail/breast-cancer) [2]. It was also ranked as the 13 th leading cause of death globally in 2021 (GBD 2021 estimation). In Italy, BC is the most prevalent cancer in women, accounting for 28–30% of all female cancers, with estimated 57,000 new cases and 13,000–15,000 deaths annually [2, 3]. Since the 1990 s, Italy has witnessed a decline in BC mortality rates among women, by 6% from 2007 to 2019 (Ministero della salute), attributed to advancements in therapy, diagnostics, and screening programs [4]. In Italy, these programs are covered by Essential Assistance Levels (Livelli Essenziali di Assistenza, so called LEA), and recommend mammograms every two years actively inviting women aged 50–69, with some regions extending the age range to 45–74. However, the COVID-19 pandemic disrupted services and reduced adherence to screening [5–7], contributing to a decline in national screening mammography rates from 46 to 30% in 2020 [3].

### Epidemiological projections and the P.I.N.K. study: prevention, imaging, network, and knowledge

Although the COVID-19 pandemic has increased epidemiological uncertainty regarding trends and forecasts of oncological incidence in general, recent projections suggest a 0.2% annual increase in BC incidence over the next two decades in Italy [8]. To address these trends and enhance prevention efforts, particularly in managing modifiable risk factors, the Italian P.I.N.K. study—Prevention, Imaging, Network and Knowledge [9, 10]—is assessing a dynamic cohort of women aged 40 and older. This multicentre study across 16 public or private diagnostic clinical centers, primarily aims to evaluate integrated imaging modalities (mammography, ultrasound, tomosynthesis, magnetic resonance, and contrast-enhanced spectral mammography) in enhancing BC detection rates and supports risk-stratified screening approaches.

### Risk-stratified screening and role of lifestyle factors in cancer prevention

Beyond the primary objective, the ultimate goal aligns with recent preventive approaches focused on risk-stratified screening. Lifestyle modifiable factors play a critical role in BC risk assessment [11, 12], and a multi-factorial approach is necessary, including both early diagnosis and participation in screening programs, as well as promotion of healthy lifestyles [13]. According to the 2018 European Commission breast guidelines [14], the risk-based screening, tailored on personal risk assessment including lifestyle features, is increasingly seen as a valid approach to improve preventive measures and early diagnosis [15, 16]. A recent systematic review showed that risk models including lifestyle parameters could help improving model performance and serve as intervention targets of prevention programs [17]. Adopting a healthy lifestyle is an effective tool in preventing cancer [16], with about 40% of cases preventable by eliminating or modifying known risk factors, such as smoking, alcohol abuse, and lack of physical activity [13], in particular in women with inherited non-modifiable risk factors [18]. Alcohol is recognized as a carcinogen by the International Agency for Research on Cancer (IARC) [19], and is one of the most frequently included modifiable factor in predictive models, in addition to non-modifiable risk factors such as genetic or reproductive ones [17]. Despite evidence linking alcohol consumption to BC risk, debates persist regarding safe consumption levels. Current data indicate increased risk even with light-to-moderate alcohol intake [20].

### Alcohol consumption and breast cancer risk in Italy

In Italy, alcohol consumption involves 58% of the population, and 17% engage in higher-risk consumption. Higher-risk alcohol consumption, reaching 13% among women, mainly concerns individuals with higher education and no economic problems (PASSI). Without aiming to develop a new predictive model, the present study aims to assess whether and to what extent alcohol consumption can anticipate an eventual breast lesion diagnosis within a convenience cohort of Italian women selected in the P.I.N.K. study (Prevention, Imaging, Network and Knowledge).

## Methods

### Structure of the data

The P.I.N.K. study (Prevention, Imaging, Network and Knowledge) is a dynamic, prospective cohort study initiated in Italy to assess breast cancer risk in women aged 40 and older. It focuses on integrating advanced imaging techniques (mammography, ultrasound, tomosynthesis, and MRI) to enhance early breast cancer detection, while also evaluating modifiable lifestyle factors. The study aims to evaluate the increased diagnostic accuracy in detecting cancers with different combinations of imaging technologies and to identify the most effective diagnostic pathway tailored to the individual patient’s characteristics, thus personalizing screening approaches. Women voluntarily and privately enroll in the study, paying for the service upon accessing the center, and are recruited on a voluntary basis. Further details on the study design can be found elsewhere [10].

A large number of women participated in the P.I.N.K. study (Prevention, Imaging, Network and Knowledge) between 2018 and 2023 (*n* = 26,572; mean age 55,7 ± 10,1; age range 40–98 years). Each participant received at least one clinical assessment and completed an auto-administered questionnaire during their first examination. For the present study, a survival cohort was selected (*n* = 3,774; mean age 58.9 ± 10.0, range 40–98 years at baseline), including women with a suspected or confirmed diagnosis of benign or malignant tumor—at any time during the study. Breast lesions were stratified into broad categories based on both biopsy report classifications (ranging from B2 to B5, lesions with uncertain malignant potential, probably malignant lesions, and positive lesions) and, where available, post-surgical BC pathology reports (*n* = 1,127). The latter provided further classification based on histological type (in situ carcinoma or invasive carcinoma), immunohistochemical expression of hormone receptors (ER/PR-positive, HER2-positive, and triple-negative), and intrinsic molecular subtypes (Luminal A, Luminal B, HER2-enriched, basal-like, and claudin-low). Nonetheless, to enhance the robustness and interpretability of the results, post-surgical BC reports were summarized into six groups: benign tumor, proliferative lesion, non-invasive tumor, invasive tumor, and mixed invasive and non-invasive tumor. Overall classification of BC lesions also included benign lesions (corresponding to B2 in biopsy reports or benign tumors in post-surgical reports), recognizing that benign tumors represent tissue changes that could be prevented and may lead to unnecessary clinical tests. Finally, the sample also included healthy women whose contributed at least 4 years to the study (*n* = 3,616). Exclusion criteria were previous positive breast biopsy or previous cancer diagnosis—any site (*n* = 877); uncertain diagnosis from cytological or micro-histological biopsy (*n* = 91); missed response to questions regarding alcohol consumption (*n* = 1).

The selection led to a convenience cohort, encompassing 3050 healthy women and 724 women with breast lesion diagnosis (see Table SM.1 in Supplementary Materials), and also referred to elsewhere as a false cohort or an available patients’ cohort, as the participants were selected based on their availability and set inclusion/exclusion criteria. The limitations of this choice are discussed in the Discussion section.

An Event History Analysis was carried out on the selected sample, as specified in the next paragraph, to estimate the time until breast lesion diagnosis (Time Ratio to event) associated with alcohol consumption levels and other selected factors. This method is used to analyze how long it takes for the diagnosis of a breast lesion to occur, tracking when the event happens over time and examining factors that might speed it up or slow it down.

Diagnosis was dichotomised as 0 for absence of lesions and 1 in case of detected lesions in the biopsy report (diagnosis from B2 to B5, including benign lesions, lesions with uncertain malignant potential, probably malignant, and positive lesions) or in the after BC-surgery report (diagnosis of benign tumor or proliferative lesion or non-invasive, invasive, or mixed invasive and non-invasive tumor type). Women with uncertain cytological or micro-histological biopsy results were excluded from the analysis. The alcohol use indicator was built based on the combination between the answers provided to the following questions: “Have you consumed alcoholic beverages in the last 12 months?” (response options: No, Yes); “If yes, how often?” (response options: less than once a week, at least once a week, several times a week, daily/almost daily); “In the last 12 months, have you had 5 or more drinks on a single occasion?” (response options: never, less than once a month, at least once a month, at least once a week, daily/almost daily). The following beverages were considered within the question about frequency of consumption: beer, wine/prosecco, alcoholic aperitifs, alcoholic cocktails, liqueurs/super-alcoholics, mix of energy drinks, and alcohol. The exposure variable was stratified in 3 levels (absent, moderate, and high alcohol consumption). It was assumed as moderate if at least one of the listed beverages was consumed once a week or less and binge drinking was never done in the last 12 months. On the other hand, it was assumed as high consumption when at least one of the listed beverages was consumed more than once a week and/or binge drinking was reported. Otherwise, the alcohol consumption was assumed as absent.

Smoking status was dichotomised in never smoker or smoker (current or former) basing on the questions “Have you ever smoked cigarettes during your life?” (response options: No, Yes) and “Have you stopped smoking currently?” (response options: No, Yes). The alcohol and smoking indicators were referred at baseline, and our model assumed the resulting pattern of consumption as prevalent for each woman over the time.

To balance the sample, family history of breast or ovarian cancer, marital status, level of education, and type of access to health care were added to the models. Family history of breast or ovarian cancer was derived from the question “Among your first-degree relatives (mother, sister, daughter), has anyone experienced a breast or ovarian tumor?” (response options: No, Yes). Marital status was built considering three categories: unmarried women, married/in civil union, and divorced/separated/widowed. Education was dichotomised as medium/low (including elementary, medium, and high school diploma) or high (bachelor’s, master’s, and postgraduate degree). Access to examination was dichotomised as public (where the costs for breast screening was covered by the National Health System) or private (where the costs for breast screening was covered by the subject).

### Event History Analysis

Event History Analysis (EHA) allows to study patterns and correlations according to which a given event occurs. The event is defined as a qualitative change of the unit of analysis, from a state *j* (non-diagnosed) to a state *k* (diagnosed), that takes place at a given point in time [21]. This allows to investigate not only the type of change, but also when it takes place, since the event assumes a preceding time interval that represents its non-occurrence [21]. By focusing on a change in outcome over time, rather than on its statistical distribution at a given point in time, the EHA is able to better analyze the processes leading to the observed outcome.

The key statistical concept of EHA is the transition (or hazard) rate, formally defined as $r\left( t \right)_{j,k} = \lim_{t\prime \to t } \frac{\Pr (t < T < t\prime |T \ge t)}{{t\prime - t}}$ where *T* is the duration before the event occurrence.

The transition rate *r*(*t*)_*j,k*_ expresses the instantaneous risk that the event occurs at time *t*, given that the event did not occur before time *t*. The EHA central concept is to use the transition rate *r*(*t*)_*j,k*_ as the dependent variable, making it dependent on a set of independent covariates and time.

Since the Kaplan–Meier estimates showed that the assumptions of proportional hazard of the Cox model were violated (analyses not reported), we opted for a log-logistic model^1^ with the Accelerated Failure Time (AFT) parameterization. This approach was chosen, since it allows for a direct interpretation of how covariates accelerate or decelerate the event occurrence, rather than assuming a constant hazard ratio over time. In fact, AFT is a method used to evaluate how certain factors (i.e., alcohol use) influence the timing of an event (i.e., breast lesion diagnosis), rather than simply determining whether these factors increase or decrease the likelihood of the event occurring. For example, if the time scale is measured in years and a continuous variable has a *β* coefficient of − 0.5, it indicates that an increase of one unit in this variable corresponds to a decrease of half a year in survival time. The AFT model, particularly with the log-logistic distribution, was deemed more suitable for our data, where the proportional hazards assumption did not hold, making it a better fit than other survival models.

The continuous time setting allowed us to use a type of statistical model called piece-wise constant exponential model [22]. In this model, the event can happen at any continuous moment in time. The origin time (*t* = 0) referring to the inception of the convenience cohort is set at 40 years of age (age of women’s entry into the P.I.N.K.—Prevention, Imaging, Network and Knowledge—cohort). The *p* values of the survival model were calculated using the *t*–test.

## Results

As reported in Table 1, high alcohol consumption showed a significantly accelerated time to event, both itself (*β* − 0.25, *p* value = 0.047) and when adjusting for non-modifiable factors (family history of breast/ovarian cancer) and social indicators (marital status, level of education, and type of access to health care) (*β* − 0.33, *p* value = 0.010).

**Table 1:** Coefficient estimates of the event history analysis model

Covariates	Reference	Level	Model 1	Model 2	Model 3
Alcohol use	Absent	Moderate	− 0.093 (0.257)	− 0.070 (0.389)	− 0.15 (0.0872)
		High	− 0.25 (0.0473)	− 0.33 (0.0103)	− 0.40 (0.00304)
Smoking	Never	Ever			− 0.035 (0.555)
Family history of breast/ovarian cancer	No	Yes		− 0.051 (0.409)	− 0.071 (0.262)
Marital status	Maiden	Married		0.36 (< 0.001)	0.36 (< 0. 001)
		Divorced/Widowed		0.29 (0. 00303)	0.26 (0.0123)
Education	Low/Med	High		0.073 (0.222)	0.071 (0.246)
Care type	Public	Private		1.46 (< 0. 001)	1.50 (< 0. 001)
Sample (n)			3,774	3,384	3,205

The event is defined as the transition from the breast lesion non-diagnosed state to the breast lesion diagnosed state. *β* coefficient and *t* test *p* value (in brackets)

Model 1: The transition from breast lesion non-diagnosed to diagnosed is assessed looking at alcohol as the only independent variable

Model 2: The transition from breast lesion non-diagnosed to diagnosed is assessed looking at alcohol as independent variable and adjusted for family history of breast/ovarian cancer, marital status, level of education, and type of access to health care

Model 3: The transition from breast lesion non-diagnosed to diagnosed is assessed looking at alcohol and smoking status as independent variables and adjusted for family history of breast/ovarian cancer, marital status, level of education, and type of access to health care

Adjustment for smoking status led to a further increase of the *β* coefficient for high alcohol consumption (*β* − 0.40; *p* value 0.003). The *β* coefficient for moderate alcohol consumption also changed after adjustment for smoking, but did not reach statistical significance (*β* − 0.15; *p*-value 0.087).

In both Models 2 and 3, family history of breast/ovarian cancer did not reach the statistical significance. The other adjusting covariates regarding marital status (married; divorced/widowed), level of education (high), and type of access to health care (private) showed a significant decelerating effect, particularly for private access to health care services.

**Fig. 1:**
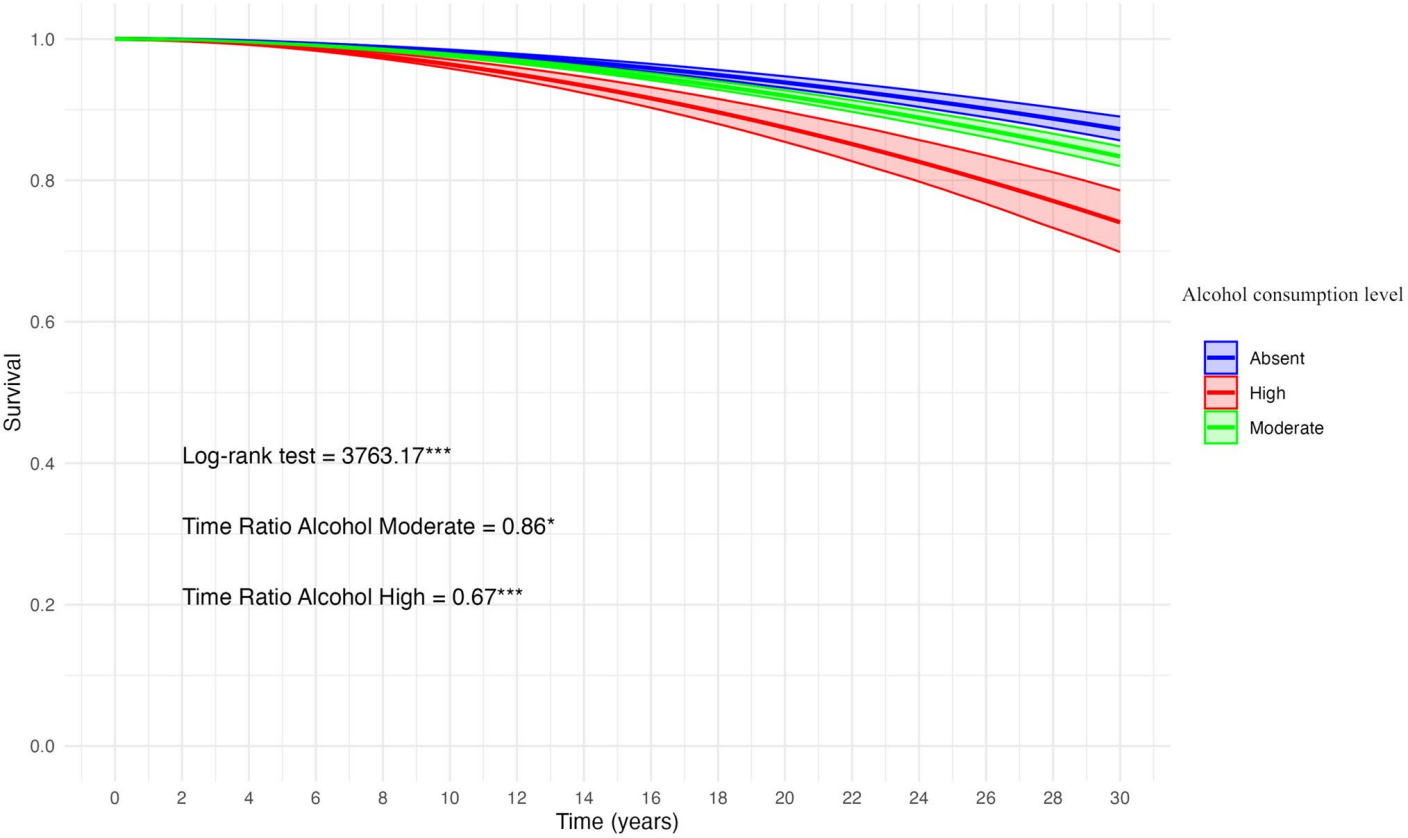
Predicted survival curve with 95% Confidence Intervals based on accelerated failure time model 3 (Table 1) for woman aged 40 + with different levels of alcohol consumption

Figure 1 shows the predicted survival curve, based on Model 3 (Table 1), related to the BC diagnosis and according to the level of alcohol consumption.

Survival model performed using the AFT parametrization with log-logistic distribution. Model 3 parameters (Table 1) used to estimate the survival function. To evaluate BC diagnosis, the model was adjusted for smoking status, family history of breast/ovarian cancer, marital status, level of education, and type of access to healthcare. Origin time (*t* = 0) corresponding to age 40. Log-rank test to assess survival curve equality. ^*^: Significant Time Ratio (TR) (*P* < 0.10); ^***^: Significant TR (*P* < 0.01).

## Discussion

A large body of research provides evidence that alcohol is a risk factor for incidence of breast cancer [23]. The World Cancer Research Fund and the American Institute for Cancer Research (WCRF-AICR) 2018 report stated that alcohol consumption is a “probable cause” and a “convincing cause” for premenopausal and postmenopausal BC, respectively, with an increased risk of 5% among premenopausal women and 9% among postmenopausal women for a 10-g increase in alcohol consumed per day (WCRF-AIRC). Our study aimed to evaluate the effect of alcohol consumption levels on the timing of eventual breast lesions onset among 3774 Italian women aged 40 years and over (mean age 58.9 ± 10.0, range 40–98 years) who visited 16 public and private centers for voluntary BC screening between 2018 and 2023. To estimate the temporal effect of alcohol consumption levels on lesion onset, we applied an Event History Analysis using Accelerated Failure Time (AFT) parameterization.

Our analysis showed that high alcohol consumption significantly accelerates lesion onset, while the effect of moderate consumption did not reach statistical significance (Model 1). After adjusting for familiality for breast or ovarian cancer, marital status, level of education, and type of access to breast examination (Model 2), high alcohol consumption remained a significant accelerating factor. Including smoking status in the model (Model 3) did not alter these findings, though high alcohol use remained the main significant accelerating factor for breast lesion diagnosis, while the anticipatory effect of moderate alcohol consumption also approached statistical significance. Smoking showed no significant anticipatory effect on breast lesion diagnosis. Indeed, this was expected because of the balanced distribution of smokers between diagnosed and not diagnosed women in our convenience sample (see SM.1 in Supplementary Materials). The predicted survival curve based on Model 3 (Fig. 1) showed a significant drop in the cumulative probability of survival to BC diagnosis in case of high alcohol consumption. For example, the 90 th percentile for high alcohol consumption corresponds to 58 years (*t* = 18), for moderate consumption to 63 years (*t* = 23), and for abstainers to 66 years (*t* = 26). Moderate alcohol consumption was found to have a time ratio that approached statistical significance, meaning that while the effect was not significant at conventional levels, it still showed a noticeable trend toward accelerating breast lesion onset. This indicates that further studies, with more participants or more granular data on consumption patterns, might be needed to confirm the effect. The results of our study fit into the broader context of risk factors associated with the development of BC, confirming that, within the observed cohort, high alcohol consumption is an important modifiable factor that can accelerate the onset of breast lesions, even after accounting for non-modifiable factors such as familiarity.

Our findings underscore the importance of including alcohol consumption in predictive models, especially in countries like Italy where alcohol consumption is culturally embedded. Furthermore, these results echo global patterns, as shown by the WHO’s recent reports highlighting the rise in alcohol-related cancer risks worldwide (WHO Global Report on Alcohol and Health 2018). Many older predictive models [24], some of which were later updated to include lifestyle factors among the predictive parameters [25], as well as recent models developed in Sweden, Spain, Cyprus, and France [26–33], do not consider alcohol consumption amid predictive parameters. There is scientific evidence that the attributable burden of disease [34] varies depending on alcohol consumption patterns, which include both volume and frequency of intake [35], and it has been hypothesized that drinking patterns may affect the risk of BC onset through modifications of insulin-like growth factor (IGF) serum levels [36]. Recent studies [37–39] provided evidence for increased risk of BC in women even due to light and light-to-moderate alcohol drinking. Given the increasing prevalence of at-risk alcohol consumption among the Italian female population, as well as the evidence linking higher alcohol consumption to greater educational attainment (Relazione al Parlamento Alcol 2021), particularly among Italian women of higher socio-economic status [40], these findings carry public health implications for targeted prevention. Data from the National Surveillance System indicate rising occasional and out-of-meal alcohol consumption among women, with 29% of 16–17-year-old girls at risk (*ISTISAN*_23*/*3_).

Our results also support the need to personalize risk-based screening programs by incorporating modifiable lifestyle factors such as alcohol use. Global data from the WHO have also emphasized the need for tailored preventive measures given varying cultural norms and consumption habits. Current universal screening approaches raise ethical concerns about equity in health access, while personalized screening can account for individual risk factors and improve early diagnosis [15, 16]. Since modifiable factors, such as lifestyle, alcohol consumption, smoking habits, as well as socio-economic status, can modify women’s personal risk, a personal assessment of these parameters could largely help in tailoring risk-based screening programs aimed at improving early diagnosis Moreover, early diagnosis has been shown to substantially reduce healthcare costs and societal burden [41]. The authors acknowledge that addressing risk-based screening programs requires considering different time windows, from menarche to the first pregnancy, from the first pregnancy to menopause, and post-menopause, allowing for a differential consideration of the hormonal status, in light of the alcohol effects on estrogen concentrations and their receptors, weight changes, and ability to metabolize ethanol by age. Furthermore, considering that familial or genetic BC typically occurs at an earlier age than sporadic breast cancer [42], and that alcohol consumption has a greater diffusion among younger women, the assessment of the biological interaction between lifestyle, genetic features, and hormonal status also has important public health implications, because it can help to identify groups of individuals who are more likely to benefit from preventive and health promotion interventions.

Despite the limitations of the study, conducted on a convenience cohort, our results confirm that among Italian women after 40 years of age, alcohol intake predicts an accelerated breast lesion onset, regardless of familiarity and socio-economic indicators. A common limitation of our study lies in the self-reported nature of the data analyzed, which may be subject to recall bias. The authors acknowledge that by selecting a convenience cohort, we may have included both women who are unfortunate enough to have already received a diagnosis, as well as those fortunate enough not to have received one yet. This selection process could have amplified the observed effect, potentially demonstrating a greater acceleration in the time to event than what might be found in a true survival cohort. Regarding the selection of women with lesions, we aimed to be as inclusive as possible due to the low number of diagnosed lesions in the total P.I.N.K. (Prevention, Imaging, Network and Knowledge) sample. For healthy women, we included those who had participated in the study for at least four years. While a narrower time window could have yielded a larger sample, we opted for a more conservative approach by including women considered healthy for a longer duration, thereby mitigating the effect of false cohort selection. The fact that this is a convenience sample, mainly from private breast centers and likely consisting of women with higher socio-economic status who can afford monitoring outside the national free screening program, does not diminish the value of our results. In fact, this may facilitate a better characterization of the target population’s risk profile. A recent study investigating the trend of Italian alcohol consumption during and after the COVID-19 pandemic highlighted an increased likelihood of at-risk alcohol consumption among women with higher socio-economic status in the long term [40]. This indicates that special preventive attention should be directed toward this at-risk sub-sample of Italian women. Another limitation of our study lies in the inability to account for alcohol consumption variables as time-varying. We recognize that this limitation necessitated the assumption that the consumption reported at baseline represented the predominant pattern throughout the woman’s lifetime. Incorporating time-varying information regarding alcohol use in future studies should certainly lead to more precise results. Moreover, given the current sample size, our dataset lacks the statistical power to support an in-depth investigation of frequency, intake, and beverage type. For these reasons, in this study, we focused on capturing the two extremes of alcohol consumption (abstinence and high consumption), which inevitably encompassed a heterogeneous range of drinking behaviors within the moderate category. This may have led to less-precise results for this group. Additionally, the decision to aggregate different types of lesions together represented a necessary choice due to the low occurrence rates observed in the sample. Additionally, a limitation of the methods used in this study is the reliance on broad stratification categories for breast lesions. Due to the complexity and variety of histological classifications in biopsy and post-surgical reports, we opted for a simplified grouping of lesions (e.g., benign tumor, proliferative lesion, non-invasive tumor, invasive tumor, and mixed invasive and non-invasive tumor) to enhance the robustness and interpretability of the results. The authors recognize that this approach does not fully capture the detailed subtypes of breast cancer [43], representing a limitation that may affect the precision of identifying subtype-specific risks. Since this study is based on a dynamic cohort, future observations will contribute to a more comprehensive analysis, allowing for a refined evaluation of the association between alcohol consumption and the risk of different types of lesions.

## Conclusion

Understanding to which extent lifestyle indicators predict risk in the current BC development models can reveal actionable targets for modification within populations engaged in high-risk lifestyles. The first general indication is that heavy alcohol use should be considered from a perspective of personalized early detection and risk-stratified screening. The study’s highlight the need for sex-sensitive approaches to address alcohol-related risks targeted interventions to promote healthier lifestyles among women. Policies could be developed to promote awareness of the risks associated with alcohol consumption and to implement community-based interventions that provide education on responsible drinking. Additionally, integrating alcohol consumption data into breast cancer risk assessment tools could inform healthcare providers and patients alike, facilitating more effective screening and intervention strategies. Implementing personalized risk-based screening programs would ensure that women at higher risk, particularly those with high alcohol consumption, receive timely and appropriate care. This approach would help promoting equity in health access and aligns with the principles of preventive medicine.

## Supplementary Information

Below is the link to the electronic supplementary material.

Supplementary file1 (DOCX 18 KB)

## Data Availability

Data are available on request from the corresponding author.

## Footnotes

1 Log-logistic function *f* (*x, α, β*) _=_^(*β/α*)(*x/α*)*β*^.^−1.^ (1 + (*x* + *α*)^*β*^)).^*2.*^

## Code availability

Codes, written totally in STATA18, are available on request from the corresponding author.

## Declarations

### Research involving human and/or animals participants

All procedures performed in studies involving human participants were in accordance with the ethical standards of the institutional and/or national research committee and with the 1964 Helsinki declaration and its later amendments.
