# Supplementary material for: Alcohol consumption and breast lesions: targets for risk-based screening in high-risk Italian women
Source: Breast Cancer. 2025 May 16;32(5):970–8. doi: 10.1007/s12282-025-01720-8 (PMC12394356; doi:10.1007/s12282-025-01720-8)
Supplement: Supplementary file 1 — Supplementary file1 (DOCX 18 KB) [file 12282_2025_1720_MOESM1_ESM.docx]

**Supplementary Materials**

**Table SM.1**: Distribution of data stratified by breast lesion diagnosis

|  | | | **Non-diagnosed** | | | | **Diagnosed** | | | |  |
| --- | --- | --- | --- | --- | --- | --- | --- | --- | --- | --- | --- |
|  |  | **n tot** | **n** | **%** | **Low** | **Upp** | **n** | **%** | **Low** | **Upp** | ***p*-value^*^** |
| **Sample** |  | 3,774 | 3,050 | 80.8 | 79.5 | 82.0 | 724 | 19.2 | 18.0 | 20.5 | <0.001 |
| **Alcohol use** | **Absent** | 539 | 449 | 14.7 | 13.5 | 16.0 | 90 | 12.4 | 10.2 | 15.0 | 0.127 |
|  | **Moderate** | 2,989 | 2,407 | 78.9 | 77.4 | 80.3 | 582 | 80.4 | 77.4 | 83.2 | 0.410 |
|  | **High** | 246 | 194 | 6.36 | 5.47 | 7.34 | 52 | 7.18 | 5.52 | 9.18 | 0.471 |
|  | **Missing** | 0 | 0 |  |  |  | 0 |  |  |  |  |
| **Smoking** | **Never** | 1,611 | 1,321 | 47.1 | 45.3 | 49.0 | 290 | 48.5 | 44.5 | 52.5 | 0.121 |
|  | **Ever** | 1,789 | 1,481 | 52.9 | 51.0 | 54.7 | 308 | 51.5 | 47.5 | 55.5 | 0.00406 |
|  | **Missing** | 374 | 248 |  |  |  | 126 |  |  |  |  |
| **Family history of breast/ovarian cancer** | **No** | 2,486 | 2,072 | 74.1 | 72.4 | 75.7 | 414 | 68.2 | 64.4 | 71.8 | <0. 001 |
|  | **Yes** | 919 | 726 | 25.9 | 24.3 | 27.6 | 193 | 31.8 | 28.2 | 35.6 | 0.119 |
|  | **Missing** | 369 | 252 |  |  |  | 117 |  |  |  |  |
| **Marital status** | **Maiden** | 507 | 405 | 13.4 | 12.2 | 14.6 | 102 | 15.4 | 12.8 | 18.3 | 0.607 |
|  | **Married** | 2,576 | 2,151 | 71.1 | 69.5 | 72.7 | 425 | 64.2 | 60.5 | 67.8 | <0. 001 |
|  | **Divorced/Widowed** | 604 | 469 | 15.5 | 14.2 | 16.8 | 135 | 20.4 | 17.5 | 23.6 | 0.0357 |
|  | **Missing** | 87 | 25 |  |  |  | 62 |  |  |  |  |
| **Education** | **Low/Med** | 2,237 | 1,785 | 58.9 | 57.1 | 60.6 | 452 | 68.1 | 64.5 | 71.5 | 0.0599 |
|  | **High** | 1,458 | 1,246 | 41.1 | 39.4 | 42.9 | 212 | 31.9 | 28.5 | 35.5 | <0. 001 |
|  | **Missing** | 79 | 19 |  |  |  | 60 |  |  |  |  |
| **Care type** | **Public** | 647 | 216 | 7.08 | 6.19 | 8.03 | 431 | 59.5 | 55.9 | 63.1 | <0. 001 |
|  | **Private** | 3,127 | 2,834 | 92.9 | 92.0 | 93.8 | 293 | 40.5 | 36.9 | 44.1 | <0. 001 |
|  | **Missing** | 0 | 0 |  |  |  | 0 |  |  |  |  |

Low: lower bound of the confidence interval; Upp: upper bound of the confidence interval

* Z-test for testing the differences between two proportions
